# A Clinical Tool for Reducing Central Nervous System Depression among Neonates Exposed to Codeine through Breast Milk

**DOI:** 10.1371/journal.pone.0070073

**Published:** 2013-07-29

**Authors:** Lauren E. Kelly, Shahnaz A. Chaudhry, Michael J. Rieder, Geert ‘t Jong, Myla E. Moretti, Andrea Lausman, Colin Ross, Howard Berger, Bruce Carleton, Michael R. Hayden, Parvaz Madadi, Gideon Koren

**Affiliations:** 1 Department of Physiology and Pharmacology, Schulich School of Medicine, University of Western Ontario, London, Ontario, Canada; 2 Department of Clinical Pharmacology, London Health Sciences Centre, London, Ontario, Canada; 3 Ivey Chair in Molecular Toxicology, University of Western Ontario, London, Ontario, Canada; 4 Department of Pediatrics, University of Toronto, Toronto, Ontario, Canada; 5 Division of Clinical Pharmacology and Toxicology, Hospital for Sick Children, Toronto, Ontario, Canada; 6 Centre for Molecular Medicine and Therapeutics, University of British Columbia, Vancouver, British Columbia, Canada; 7 Child and Family Research Institute, Vancouver, British Columbia, Canada; 8 Department of obstetrics and gynecology, St. Michael’s Hospital, Toronto, Ontario, Canada; The Ohio State Unversity, United States of America

## Abstract

**Background:**

Neonates are commonly exposed to maternal codeine through breast milk. Central Nervous System (CNS) depression has been reported in up to 24% of nurslings following codeine exposure. In 2009, we developed guidelines to improve the safety of codeine use during breastfeeding based on previously established pharmacogenetic and clinical risk factors. The primary objective of this study was to prospectively evaluate the effectiveness of these guidelines in ensuring neonatal safety.

**Methods and Findings:**

Women taking codeine for pain following caesarean section were given safety guidelines, including advice to use the lowest codeine dose for no longer than four days and to switch to a non-opioid when possible. Mothers provided a saliva sample for analysis of genes involved in opioid disposition, metabolism and response. A total of 238 consenting women participated. Neonatal sedation was reported in 2.1% (5/238) of breastfeeding women taking codeine according to our safety guidelines. This rate was eight fold lower than that reported in previous prospective studies. Women reporting sedated infants were taking codeine for a significantly longer period of time (4.80±2.59 days vs. 2.52±1.58 days, p = 0.0018). While following the codeine safety guidelines, mothers were less likely to supplement with formula, reported lower rates of sedation in themselves and breastfed more frequently throughout the day when compared to previously reported rates. Genotyping analysis of cytochrome p450 2D6 *(CYP2D6*), uridine-diphosphate glucuronosyltransferase (UGT) 2B7, p-glycoprotein (*ABCB1*), the mu-opioid receptor (*OPRM1*) and catechol-o-demethyltransferase (*COMT)* did not predict codeine response in breastfeeding mother/infant pairs when following the safety guidelines.

**Conclusions:**

The only cases of CNS depression occurred when the length of codeine use exceeded the guideline recommendations. Neonatal safety of codeine can be improved using evidence-based guidelines, even in those deemed by genetics to be at high risk for toxicity.

## Introduction

Codeine-containing medications are commonly used for postoperative pain following caesarean section or episiotomy [1]. Maternal breast milk is the ideal feeding method for newborns and is recommend by The American Academy of Pediatrics [2] and the World Health Organization [3]. In Canada, up to 80% of new mothers initiate breastfeeding [4] and between 20–33% of all births are by caesarean section, rendering thousands of newborns exposed to codeine, and its active metabolites, morphine and morphine-6-glucuronide (M6G) through breast milk [5] [6].

Codeine is often considered a pro-drug, as the majority of its analgesic properties result from its biotransformation into morphine by the highly polymorphic cytochrome P450 enzyme 2D6 (*CYP2D6*). Morphine is further metabolized to the active morphine-6-glucuronide (M6G) and inactive morphine-3-glucuronide by UDP-glucuronosyltransferase (UGT) 1A1 and 2B7. The active metabolites of codeine, morphine and M6G, relieve pain via their action at the mu-opioid receptor. *CYP2D6* has over 80 variant alleles which differ in their contribution to enzyme activity [7]. Individuals with a functional gene duplication, resulting in the ultra-rapid metaboliser (UM) phenotype, have on average 50% higher plasma concentrations of morphine and M6G than the wild-type [8]. The CYP2D6 UM phenotype in mothers has been associated with an increased risk for neonatal central nervous system (CNS) depression (9). Conversely, two alleles with no activity results in a poor metabolizer (PM) phenotype and these patients typically receive little or no therapeutic benefit from codeine as they form negligible amounts of the active metabolites [7] [8]. Polymorphisms in drug transport by p-glycoprotein (*ABCB1*), the opioid receptor (*OPRM1*) and catechol-o-demethyltransferase (*COMT*) are also thought to cause variability in response to codeine, morphine and their metabolites.

**Figure 1:**
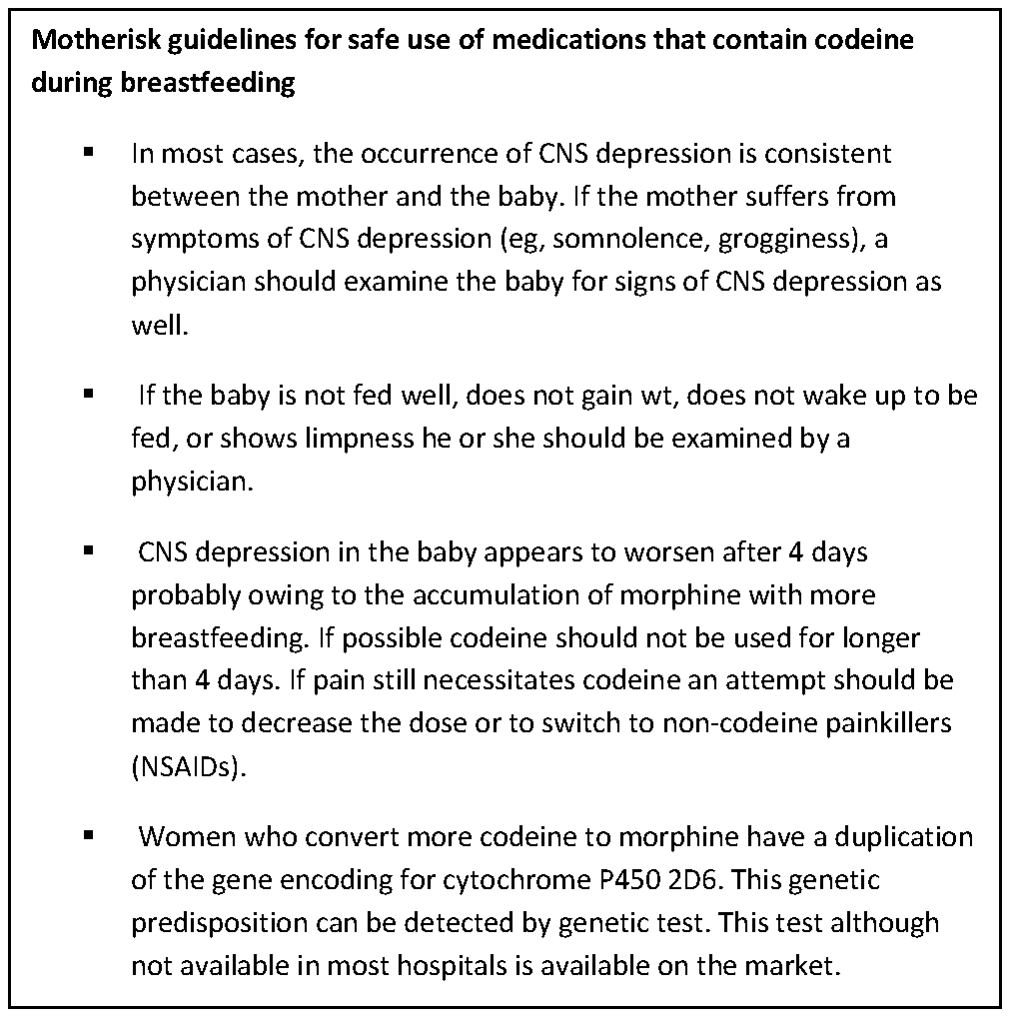
Safety guidelines for codeine use during breastfeeding. Motherisk safety guidelines for codeine use in breastfeeding pamphlet given to all expectant mothers with planned caesarean sections at St. Michaels Hospital in Toronto, Ontario, Canada (14). Women were advised to take codeine for as short a period as possible (3–4 days postpartum) and were advised to seek the care of a physician if they required pain medication beyond this point. Mothers were also advised to breastfeed before taking codeine to maximize the time to eliminate codeine in between feeds. ©Motherisk Program and The Hospital for Sick Children. Reprinted with permission from the Canadian Family Physician.

In 2006, our group reported a fatal case of CNS depression in a breastfed infant resulting from morphine overproduction in a CYP2D6 UM mother [10]. Following the publication of this case, the United States Food and Drug Administration as well as Health Canada published public health advisories and label changes warning that codeine may not be safe during breastfeeding for infants whose mothers are CYP2D6 UMs due to an increase in morphine production [11] [12]. Since then, a prospective study of women taking codeine postpartum revealed that 16.7% (35/210) of mothers reported CNS depression in their infants following postpartum use of codeine [13]. These risks of neonatal CNS depression led the Motherisk program to critically evaluate the available scientific evidence and propose guidelines for safe use of codeine during breast feeding [14]. Further pharmacokinetic data confirmed that potentially toxic morphine concentrations can be reached within 4 days in breastfed neonates after multiple doses of maternal codeine [15]. The repeated observations of CNS depression after more than 4 day exposure to codeine through milk, the high concordance between maternal and neonatal CNS depression, and the typical adverse symptoms; including prolonged sleep, missing feeding, poor latch and poor weight gain were identified as clinical factors associated with infant CNS depression following codeine exposure (Figure 1) [14]. The primary objective of the present study was to evaluating the effectiveness of these safety guidelines at improving neonatal safety.

## Patients and Methods

This study was approved by the institutional Research Ethics Board at the University of Western Ontario and St. Michael’s Hospital. Mothers taking codeine for pain following caesarean section at St. Michael’s Hospital, Toronto, Canada were recruited between December 1, 2009 and November 30, 2011. A team member obtained written and informed consent from the expectant mother, as well as provided all study information and answering questions. At the time of their post-operative codeine prescription, women were provided with a copy of the codeine safety guidelines (Figure 1). At the time of recruitment, mothers were advised rregarding mode of action of codeine, effect of genetic makeup of mother on conversion of codeine to morphine. They were also informed about the possible adverse effects in mothers like sedation, dizziness, drowsiness, and constipation. Information was also given regarding the possible adverse effects on the breastfed neonates such as poor latching onto the nipple or poor feeding, limpness, prolonged sleep or difficulty breathing. Mothers were given a 24 hour contact telephone number and were advised to bring their child to an emergency department if any of the above symptoms were noted. Due to the previously high reported rates of neonatal sedation 16% (35/210) it was deemed unethical to randomize a control arm without educational intervention [13].

Following delivery, provided it was unremarkable, the mother and infant normally remained in hospital for several nights before being discharged home. In the 16-hour period after delivery the mother was not typically given any analgesic medication except for epidural analgesia. After 16 hours most mothers began taking Tylenol #3 (500 mg acetaminophen and 30 mg codeine) for their pain every 4–6 hours as needed and were sent home with a prescription for this medication or other non-opioid analgesic(s). All mothers were given a “Patient Medication and Breastfeeding Tracking Sheet” to track breastfeeding progression, medication use and infant health. One week after discharge, a follow-up telephone interview was conducted to assess maternal and infant health with a specific focus on neonatal CNS depression. Follow-up was conducted using a standardized questionnaire to ensure all participants provided the same quantity and quality of follow-up data. In order to identify neonatal sedation data was collected on the several parameters including the number of times an infant feed per night, whether or not they woke up for feeds and the length of time per feed. At the time of recruitment mothers were advised to look for any latching problems, breathing difficulties, or any fluctuations in skin colour which was reported at the time of follow-up. Data was also collected on the number of bowel movements per 24 hours, the length of sleep at one time and the total amount of sleep per night. The interview also included maternal satisfaction with pain control, self-reported analgesic dose, and any maternal adverse drug reactions.

Mothers had the option of providing a saliva sample for genetic screening. The saliva sample was labelled with a unique barcode and couriered to the Canadian Pharmacogenomic Network for Drug Safety (CPNDS) Core Laboratory in Vancouver, British Columbia. In order to determine the role of genetic variability on the codeine and morphine pathways, polymorphisms in several key genes (ABCB1, COMT, UGT 2B7, OPRM1) were analysed. Maternal DNA was purified from saliva samples using the QIAmp DNA purification system (Qiagen, Toronto, ON, Canada) according to the manufacturer’s protocol. DNA samples were genotyped for variants in CYP2D6 using the AutoGenomics INFINITI® Analyzer and the CYP450 2D6I Assay (AutoGenomics Inc., Vista, CA, USA), as well as SNaPshot, and TaqMan copy number assays as previously described [16]. A genotype activity score was calculated based on the scores of the individual alleles and patients were classified into four *CYP2D6* phenotype classes: poor metabolizers (PM: activity score of 0); intermediate metabolizers (IM: activity score 0.5–1); extensive metabolizers (EM: activity score 1.5–2; and ultra-rapid metabolizers (UM: activity score >2 due to a functional gene duplication) [21]. CYP2D6*1 or *2 alleles were assigned an activity score of 1, the partially functional *9, *10, *17, *29, or *41, alleles were assigned an activity score of 0.5, and the non-functional *3, *4, *6, *7, *8, *12, or *14 alleles were assigned an activity score of 0. Whole gene deletions (*5) were assigned a score of 0. Activity scores in the case of whole gene duplications were assigned according to the number of functional *CYP2D6* copies. If none of the aforementioned alleles were detected, the individual was assigned the default wild-type allele of *1. Gaedigk and colleagues describe in more detail the *CYP2D6* alleles [17].

DNA samples were also genotyped for genetic variations in the ABCB1 gene: 61 A>G (rs9282564), 1236C>T (rs1128503), 2677G>T/A (rs2032582), and 3435C>T (rs1045642), COMT (rs165815, rs4633, rs4818, rs740602) and OPRM1 gene 118 A>G (rs1799971) using TaqMan® genotyping assays (Applied Biosystems, Foster City, CA, USA), as previously described [16].

Continuous parametric data were compared using an unpaired Students t test and are reported as mean values with the corresponding standard deviation. Nonparametric continuous data are reported as median (range) where appropriate and were analysed using the Mann Whitney U test. Normally distributed data were compared by Student’s t test for unpaired results. All discrete and binary data is conveyed in percentages and significance was tested using a Fisher Exact test.

## Results

A total of 255 women consented to participate in this study, however 15 were lost to follow-up and one woman withdrew for personal reasons. There were 268 women approached to participate in the study. We included 238 women giving birth to 239 healthy babies (129 males, 108 females and one set of male/female twins). Ethnicity was self-described, and the mother`s grandparent’s country of origin were documented. There were 87 women that identified as Caucasian (37%), 42 as South or Central American (18%), 36 as Asian (15%), 34 as African American (14%), 24 identified as Indian (10%) and the remaining 15 originated from other countries. Saliva samples were collected from all participants.

Expectant mothers’ mean weight was 74.86+/−12.10 kg prior to delivery with a mean age of 32.82+/−5.58 years. Following delivery mothers remained in hospital for a mean of 1.91+/−0.75 days. The mean dose of codeine taken by the participants was 1.18+/−0.54 mg/kg/day for 2.56+/−1.51 days. When asked why they stopped taking codeine, 86% (204/237) reported that they had no more pain, 7% of mothers that stopped taking codeine due to adverse reactions in themselves (10/237 for excessive sedation, 6/237 for constipation,). Six mothers, (2.5%) discontinued codeine use over concerns for their infants and 4.6% stopped taking codeine because they found it ineffective in managing their pain. Patient’s genotype did not correlate with the reasons for codeine discontinuation. There was only one mother who contacted the 24 hour hotline.

Five women reported sedation in their child following exposure to codeine in breast milk (2.1%, 5/238). None of these five mothers reported adverse reactions to codeine in themselves during use of the drug. There were no significant differences identified in the length of time spent breastfeeding, or the total amount of codeine used (Table 1). Women reporting sedated infants were taking codeine for a significantly longer period of time, and on average (4.80 days) in excess of the 4 days recommended by the guidelines. (4.80±2.59 days vs. 2.52±1.58 days, p = 0.0018 by Students t test). There were no sedated infants or maternal adverse drug reactions reported in the CYP2D6 ultra-rapid metabolizer group. Mothers with CYP2D6 UM status breastfed for a similar length of time and took similar amounts of codeine as all remaining phenotypes (Table 2). A total of 9% (19/238) of mothers reported adverse reactions following codeine use postpartum. These complaints included sedation, dizziness, constipation and nausea. Maternal adverse response did not correlate with neonatal sedation (0/19 vs. 5/219). All mothers with symptomatic neonates were non-Caucasian in origin.

**Table 1:** The influence of clinical factors on neonatal sedation (Infant ADR).

	(N = 5)	(N = 233)	
	Infant ADR	Asymptomatic	P Value
No. days taking codeine	4.8±2.6	2.5±1.6	0.01
Total amount of codeine used (mg/kg)	5.1±2.4	3.2±2.5	0.08
No. times feeding per day	8.5 (7.0–9.0)	9.0 (3.0–13.0)	0.27
Baby GA (weeks)	38.6±1.1	39.1±1.4	0.44
Baby Birth Weight (g)	3392.4±584.4	3403.1±510.12	0.96
No. consecutive hours slept	3.5 (2.5–4.0)	2.5 (1.5–4.5)	0.19
Maternal ADR	0	8% (19/242)	0.67
Formula supplementation	20% (1/5)	17% (39/229)	0.41
Non-Caucasian	100% (5/5)	62% (143/230	0.10

Demographic characteristics of Mothers of sedated infants (Infant ADR) and those whose infants were did not report any changes in health status (asymptomatic). Statistical significance was set at P<0.05. Parametric values are presented as mean ± standard deviation and nonparametric values are presented as median (range).

**Table 2:** The influence of CYP 2D6 genetic factors and breakdown of demographics.

	Poor Metabolizer(N = 11)	Intermediate Metabolizer(N = 70)	Extensive Metabolizer (N = 105)	Ultra-rapid Metabolizer (N = 6)
Total Codeine Dose (mg/kg/day)	1.4±0.6	1.1±0.4	1.2±0.5	1.2±0.3
Total No. days on codeine	2 (1–3)	2 (1–9)	2 (1–9)	2 (1–3)
Maternal Age (years)	34.5±5.5	32.9±5.7	32.6±5.51	32.0±6.8
No. times breastfed in 24hours	9 (7–10)	9 (6–13)	8.75 (3–11.5)	9 (7–13)
Baby GA (weeks)	39.3±1.4	39.0±1.2	38.5±1.3	38.8±0.4
Baby weight (grams)	3517.5±513.4	3379.4±510.8	3421.3±519.5	3199±321.8
No. reported infant ADRs (%)	0	3 (4%)	2 (2%)	0
No. reported maternal ADRs (%)	1 (9%)	7 (10%)	7 (6%)	0

Patient characteristics and reported outcomes broken down by CYP2D6 phenotype, where a poor metabolizer has a CYP2D6 activity score of 0, intermediate a score between 0.5–1.0, extensive a score between 1.5–2.0 and ultra-rapid a score of 2.5 or higher. Data are presented as mean ± standard deviation, and median (range) for nonparametric data.

Maternal DNA was analyzed for polymorphisms in ABCB1, COMT, UGT 2B7, OPRM1. Maternal adverse events did not correlate with any of the genes analysed in this study (Table 3). Furthermore there were no associations of infant sedation with any of the genes evaluated. The minor allele frequency (MAF) for all polymorphisms was not significantly different from the global reported MAF as taken from the NCBI dbSNP database (http://www.ncbi.nlm.nih.gov/projects/SNP/).

**Table 3:** The influence of genetic factors on reported maternal adverse effects.

Gene	SNP			Global Minor AlleleFrequency (MAF):	Observed MAF in allstudy participants (N)	Allele		Percent of mothersreporting ADRS (N = 19)	Percent of healthy mothers (N = 218)		P Value		
**ABCB1**	rs1128503			0.42	T = 0.445 (212)	**TT**		**21**	**25**		0.21		
						**TC**		**37**	**44**		0.16		
						**CC**		**42**	**31**		0.12		
	rs2032582			0.34	A = 0.21 (100)	**AA**		**21**	**16**		0.19		
					T = 0.21 (98)	**AT**		**5**	**6**		0.38		
						**GG**		**32**	**39**		0.16		
						**GT**		**37**	**35**		0.19		
						**GA**		**5**	**3**		0.38		
	rs1045642			0.40	T = 0.44 (207)	**TT**		**26**	**21**		0.19		
						**TC**		**32**	**45**		0.10		
						**CC**		**42**	**34**		0.15		
**COMT**	rs4680			0.39	A = 0.43 (204)	**AA**		**21**	**21**		0.23		
						**AG**		**32**	**46**		0.10		
						**GG**		**47**	**33**		0.10		
	rs4633			0.39	T = 0.42 (200)	**CC**		**47**	**34**		0.10		
						**CT**		**37**	**45**		0.15		
						**TT**		**16**	**21**		0.27		
	rs4818			0.32	C = 0.65 (306)	**CC**		**37**	**41**		0.18		
						**CG**		**42**	**48**		0.17		
						**GG**		**21**	**11**		0.12		
**OPRM1**			rs1799971	0.19	G = 0.22 (105)		**AA**	**68**		**62**		0.18	
						**AG**		**32**	**30**		0.20		
						**GG**		**0**	**8**		0.23		
**UGT 2B7**		rs7439366		0.47	T = 0.44 (206)	**CC** **CT** **TT**		**37** **37** **36**	**36** **40** **24**		0.200.190.21		

Minor allele frequency for polymorphisms in the p-glycoprotein transporter (ABCB1), cathechol-o-methyltransferase (COMT), mu-opioid receptor (OPRM1) and UDP glucuronosyltransferase (UGT) 2B7 in mothers reporting adverse drug reactions (ADR) in themselves compared to those that were asymptomatic (healthy). Significance value was set at P<0.05.

## Discussion

Given the considerable genetic variability in codeine response, its frequent use postpartum, and the previously reported high rates of neonatal sedation, it was imperative to evaluate the effectiveness of a clinical tool to improve infants’ safety. We have developed safety guidelines that combined clinical data from two case control studies with previously identified genetic markers of toxicity [9] [11] [13]. The present study is the first prospective study to evaluate the effectiveness of codeine guidelines for breastfeeding mother/infant pairs. The safety guidelines developed recommend use of codeine for no longer than four days postpartum, as generally milk production is lower in these first few days of life, and thus neonatal morphine accumulation is minimized [14] [15]. The only significant difference seen between symptomatic and nonsymptomatic infants in the present study was the duration of codeine use, where neonatal CNS depression occurred among infants exposed to codeine for more than 4 days. These data suggest that minimizing codeine use to the first four days can significantly reduce neonatal exposure and reduce the incidence of reported neonatal CNS depression.

Based on current codeine warnings, ethical considerations precluded the randomization of a group of mother-child pairs to not receive counselling on the potential neonatal risks of codeine use thus preventing a true control group. Using the Motherisk Codeine Safety Guidelines [14], the present cohort reported considerably lower rates of neonatal sedation [2% (5/238)] than a previously reported rate in a prospective study [16% (35/210)] [13]. The lack of formal randomized control arm in this trial does introduce some limitation in data interpretation as selection criteria and the time of follow-up is variable between studies. When following the guidelines mothers were less likely to supplement infant feeding with formula (16.74%) which was reported at 57.06% in a previously prospective cohort of women breastfeeding while taking codeine post-caesarean section [13]. Providing mothers with the education tools to make informed decisions regarding their choice to breastfeed is important as maternal breast milk provides optimal nutrition for neonates.

This study further supports educating mothers on the symptoms of codeine toxicity in their infants and the importance of a prompt response if these symptoms appear. The guidelines also recommend that if a woman is still in pain after 4 days of codeine use, she should attempt a non-opioid analgesic such as ibuprofen [14]. In this study 86% of women reported that they discontinued codeine because their pain was well managed before 4 days. This suggests that codeine is effective and safe in treating maternal pain following caesarean section when used according to the Motherisk guidelines for no more than four days.

There are several genetic polymorphisms important in determining codeine and morphine response. Previous work has identified maternal genetic factors that increase the risk for sedation in neonates exposed to codeine through breast milk including CYP 2D6 UM phenotype, UGT 2B7*2/*2 and *ABCB1* 2677TT [9] [18]. The UGT 2B7 C802T variant which gives rise to a *UGT 2B7* *2 phenotype has shown to increase production of M6G, which is several fold more potent than morphine [19]. A large portion of the original codeine dose is also glucuronidated by *UGT2B7* into codeine-6-glucuronide. This polymorphism was associated with an increased risk for neonatal sedation when found in combination with the CYP2D6 UM phenotype; however this finding has not been repeated [9]. The increased morphine production seen with the CYP 2D6 UM phenotype has been associated with an increased risk for codeine related adverse events in both mother and infant [9]. Of importance, while following the safety guidelines, there were no maternal or neonatal adverse events associated with the CYP 2D6 or UGT 2B7 genotype (Table 2). There is ethnically determined variability in polymorphism frequency that should be evaluated in future studies. For example, the frequency of the CYP 2D6 UM phenotype has been reported to be as high as 40% in women of North African descent, which is only described in approximately 2% of Caucasians [23]. In this cohort none of the mothers of symptomatic infants conveyed the CYP 2D6 UM phenotype, however, all five women identified themselves as non-Caucasian. While it is important to independently study the effects of ethnicity on codeine response, the present cohort represents the diverse patient populations typically seen in many North American Institutions.

The genetic polymorphisms responsible for the production of morphine are not the only sources responsible for the variability seen in codeine response. Single nucleotide polymorphisms (SNP) in P-glycoprotein (ABCB1) have been associated with increased central adverse effects in patients taking morphine [20]. This efflux transporter protein plays an important role in transporting codeine and morphine out of the brain. The maternal ABCB1 2677TT polymorphism is significantly associated with neonatal CNS depression following breastfeeding resulting from a decrease in efflux out of the blood brain barrier [18]. Conversely, a seemingly protective SNP in the mu-opioid receptor (OPRM1) A118G decreases receptor expression results in a lower incidence of opioid toxicity [21]. Catechol-o-methyltransferase (COMT) is responsible for the breakdown of neurotransmitters and interacts with the opioid receptor. Increased morphine sensitivity has been associated with several COMT SNPs [22]. Polymorphisms in maternal OPRM1 and COMT have not be associated with an increased risk for neonatal sedation following breastfeeding but their importance for morphine effectiveness warrants further investigation. Currently these polymorphisms are not routinely screened for before a patient is given codeine for pain.

Our study shows that post-partum safety guidelines can improve the safety of codeine exposure in virtually all breastfed infants regardless of their genetic profile. Alternatively, post-partum maternal analgesia with Nonsteroidal anti-inflammatory agents (NSAIDs) should also be considered, as a recent systematic review of all randomized trials suggests that codeine is not superior to NSAIDs for analgesia after laparotomy [24]. In conclusion, in our study mothers following the safety guidelines reported low levels of neonatal sedation (2%). Maternal adverse event rates were low, even in those genetically determined to be of high risk while maternal pain was effectively managed. These guidelines present a simple and effective approach to improving the neonatal safety while maintaining effectiveness of postpartum codeine use.
